# The Effect of Floorball Training on Health Status, Psychological Health and Social Capital in Older Men

**DOI:** 10.3934/publichealth.2017.4.364

**Published:** 2017-07-10

**Authors:** Johan M. Wikman, Anne Nistrup, Jacob Vorup, Mogens T. Pedersen, Pia S. Melchor, Jens Bangsbo, Gertrud Pfister

**Affiliations:** Department of Nutrition, Exercise and Sports, University of Copenhagen, Copenhagen, Denmark

**Keywords:** aging, ball games, indoor hockey, old age, quality of life, social network

## Abstract

This article presents the results of a multidisciplinary study which investigated the effects of a period with floorball training on health status, psychological health and social capital of older men. Thirty-nine untrained men aged 69.9 ± 0.6 (range: 65–76) were randomized into a group playing floorball (n = 22) or a group playing petanque (n = 17) one hour twice a week for 12 weeks. Both groups filled out the Health Survey Short Form (SF-12) and the Hospital Anxiety and Depression Scale (HADS) before and after the 12-week intervention. Linear regression analyses with bootstrapping showed that the men in the floorball group improved in the SF-12 composite score for mental health, as well as the HADS subscales anxiety and depression, compared to the men in the petanque group. In addition, 21 interviews were conducted with a sample of the men engaged in floorball. According to the statements in the interviews, the men in the floorball group experienced a high degree of solidarity and group cohesion which seemed to have increased their social capital during the intervention. In particular, the fun and joyful experiences of playing led to a high degree of social connectedness, which were mentioned by many of the men as the main reason for their participation throughout the 12-week period. The statistical results and the interview findings suggest that participation in a ball game such as floorball has several benefits regarding health status, psychological health and social capital and in addition that playing floorball is experienced as enjoyable amongst older men. Thus, it can be concluded that floorball is an activity that benefits older men and should be provided in relevant contexts, such as e.g. sport clubs or centres for seniors.

## 1. Introduction

Aging can have many noticeable consequences for physical and psychological health. Among these consequences is physiological decline, such as accumulation of body fat [1] as well as loss of functional capacity and muscle strength [2], together with several geriatric syndromes [3]. Furthermore, the prevalence of mental disorders, such as anxiety [4], depression [5] and dementia [6], is considerably higher among the older population than among younger age groups. In addition, the amount and quality of social relations can decrease with age [7]. Negative consequences of social isolation can be physical or functional decline [8]–[10], as well as decline in psychological health or cognitive functioning [4],[5],[9],[10]. It seems that old age make people prone to deterioration both in physical and psychological health.

However, there are possibilities to prevent or diminish the negative consequences of aging. Participation in physical activity seems to be a possible way to alleviate the physical decline that is often connected aging [11],[12]. To this end, team games have been shown to produce positive cardiovascular effects due to the intermittent nature of the activity [13],[14], and can produce health benefits in older adults that are similar to those produced by resistance training [15].

The decline in psychological health can be alleviated as well. It is commonly accepted that physical activity has positive effects on psychological health (e.g. [16],[17]). Furthermore, interventions conducted with groups can be an effective remedy for social isolation and loneliness [18]. For example, Pedersen et al. [15] found that both resistance training in groups and team games improved participants' psychological health and quality of life. It seems that participation in physical activity in general and in group activities seems beneficial for physical and psychological health.

Team games may be an additional remedy for the decline in physical and psychological health. Regular small-sided football and floorball training among untrained men and women seems to bring about important health benefits [14],[15],[19],[20]. In addition, team games seem to be motivating, due to their interactive and social nature [15],[21] and the element of play, which is a key motivating factor that is not present in individual fitness activities [21]. In addition, it seems that the team activities can give participants something to look forward to and belongingness to a group, which can decrease social isolation [15]. Nielsen et al. [21] found that older men who participated in football gained improved quality of life and a social network on the team. Their findings are similar to the results of Ottesen, Jeppesen, and Krustrup [22], who also found that participants developed social capital from the group activities. In a Bourdieusian framework, social capital is a resource that is connected to group membership and social networks, where an individual's effort to improve his/her social position in a variable of different social arenas or “fields”, arise from developing social relations. Bourdieu emphasizes conflicts and the operation of power, whereby an individual can advance her/his interests within a social arena [23].

The above mentioned benefits of participation in team games are of particular importance to older men. Although participation in sports and physical activity is relatively high among Danes aged 60 years or older, men in this age group are less physically active than women (58% of men aged 60–69 and 56% aged 70 and above are physically active, compared to 73% and 66% of women) [24]. The lower level of participation in sports can be caused by the fact that men are not part of communities where sports and exercise are practiced, or simply; a higher proportion of men experience a lack of motivation to sport and exercise and may feel that the exercises offered are not interesting and motivating [25]. Furthermore, it seems that some men find it hard to enter the physical activity context, as they find it excluding to newcomers [21]. The consequence is that many men do not benefit from physical activity and its associated social relations, which support resistance against the negative physical and psychological consequences of aging [26]. Therefore, to participate in physical activity, it is particularly important for older men to be offered a motivating activity that is offered in an including context. It appears that team games can meet these expectations [15],[21], and it seems that team games can be a fruitful way to alleviate some of the decline in psychological health that is brought about by old age in men. However, we do not know whether the social interaction embedded in a team game is the primary cause of an improvement in psychological health, or if the physical activity combined interaction during the activity adds to this.

To find the answers to this question, the overall purpose of the study was to investigate the effects of a team game, namely floorball training, on health status and psychological health compared to a control group participating in an a team game with little or no physical activity, namely petanque, in a group of untrained men aged 65 years or older. In addition, we wanted to investigate possible reasons for the hypothesized difference between the effects of participation in floorball and petanque.

## 2. Methods

This study was an integrated part of a multidisciplinary intervention, which also included physiological investigations. For more details on the study and the results, see Vorup et al. [14]. The study was approved by the local ethics committee of Copenhagen (H-2-2013-149) and conducted in accordance with the guidelines of the declaration of Helsinki. The subjects were informed of any risks and discomforts associated with the study before giving their written informed consent to participate in the study.

### 2.1. Participants

**Figure 1.**
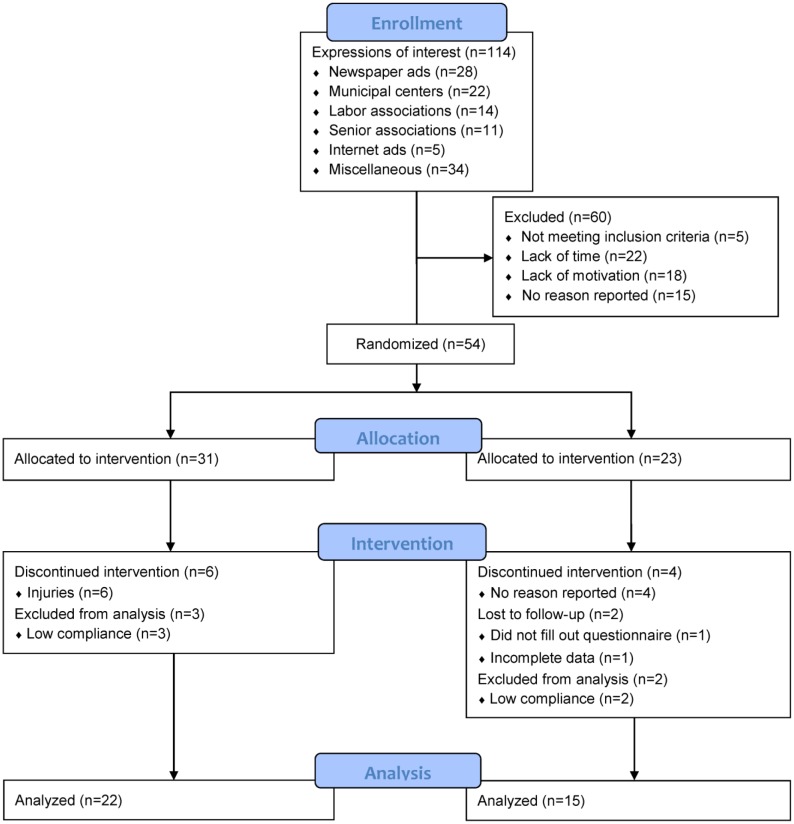
Participant flowchart.

Thirty-seven untrained men aged 69.84 (SD = 3.66, range: 65–76) years were recruited (see flowchart in Figure 1).

The men were recreationally active (walking or cycling for transportation on a daily basis, and some gymnastic and swimming activities once per week), but had not been involved in any type of regular (<2 weekly sessions) intense physical training for at least 10 years. Over two intervention periods, the men were randomized into a floorball group (n = 22) or a petanque group (n = 15). The reason for the unequal group sizes is that in the first intervention period, the participants were randomized 1:1, but in the second, they were randomized 2:1. This was a decision made by Vorup et al. [14] due to their primary interest in the effects of the floorball training.

### 2.2. The intervention

Both groups met twice per week to participate in floorball or petanque, respectively, and adherence was 64.64% (SD = 10.49) for floorball and 64.74% (SD = 12.88) for petanque.

#### 2.2.1. Floorball training

Floorball is a team sport similar to hockey, but is played with plastic sticks (floorball.org). The training was arranged as small-sided games, e.g. 3 vs. 3 or 4 vs. 4, on in- or outdoor courts sized 10–15 × 20–30 meter. The training sessions were organized as intervals with 4 min of playing followed by 4 min of rest with 3 intervals during the first four weeks (=12 min “active play” in each session), 4 intervals during week five to eight (= 16 min “active play” in each session), and 6 intervals in week nine to twelve (= 24 min “active play” in each session). Each training session was preceded by 10 min warm-up, which included mobility, stretching and technical exercises.

#### 2.2.2. Petanque activity

Petanque is a traditional team sport game, in which two teams compete to throw their balls as close to a smaller ball as possible (petanque.org). It offers little physical activity or contact. The training was organized as small-sided games, typical 2 vs. 2 or 3 vs. 3, and was performed outside in a park area. Each session has a total duration of 60 minutes, and in case of bad weather conditions, the men played billiard inside.

### 2.3. Questionnaire data

#### 2.3.1. Questionnaires

To measure health status and psychological health, the following questionnaires were distributed to the men before and after the intervention.

The Danish version of the Health Survey Short Form (SF-12 [27]) was used to measure health status. It contains 12 questions that can be summarized into two composite scores, one for physical and one for mental health [28]. The SF-12 has been validated for the sample and produce valid results comparable with the longer version, the SF-36 [27].

A Danish version of the Hospital Anxiety and Depression Scale (HADS [29]) was also used to assess psychological health of the men. It contains two subscales measuring anxiety and depression. Each subscale contains seven questions, with a Likert-scale answering format from 1 to 4, whose end points differ depending on each question. The questionnaire has been validated in Danish.

#### 2.3.2. Statistical analyses

##### (1) Group differences before the intervention

To test whether the groups had the same vantage point before the intervention, group differences in age, as well as all subscales of SF-12 and HADS before the intervention were tested with a Mann-Whitney test.

##### (2) Intervention effects

To test effects of the training intervention, linear regression analyses with bootstrapping were used, in which measurements of the SF-12 composite scores for physical and mental health and the HADS subscales anxiety and depression at the end of the intervention were used as dependent variables, and measurements before the intervention and group affiliation (floorball or petanque) were used as independent variables.

### 2.4. Interviews

To gain insight into the men's subjective experiences of participating in floorball training, 21 individual interviews were conducted with the men in the floorball group one to three weeks after the finalisation of the intervention. To avoid bias, for example from socially desirable answers, these interviews were conducted by a researcher who had no prior involvement with the participants.

#### 2.4.1. Observations

Prior to the interviews, participant observations were conducted with the purpose of establishing positive relationships to the men in an informal manner and gaining insight on how the floorball sessions were conducted. The observations and reflections were written down as field notes and influenced the development of themes in the interview guide as a supplement to the theoretical framework [30]. Interaction on and off the pitch as well as how the men built social networks [31],[32] arose as the main focus points from the observations. The observations were used in order to add another dimension to the collected interviews and to get insight into how the participants acted and interacted, in contrast to what they said [33].

#### 2.4.2. The interview guide

The interview guide was influenced by the participant observations of the training sessions during the intervention. As a theoretical framework, Bourdieu's concept of habitus and social capital, which provides insight and understanding of the social relationships between individuals [23], was used. The focus of the interviews was related to the men's everyday lives and their reasons for and barriers to participate in physical activity, as well as their previous experiences with physical activity and team sport in particular. The aim of the interviews was to investigate the men's experiences with floorball training, and how these experiences were related to their life biographies, general health behaviour, previous sport experiences, and motivation to physical activity in general.

#### 2.4.3. Conducting the interviews

The interviews were based on a semi-structured interview guide [34], and were conducted in a rather informal atmosphere. This enabled the interviewer to follow up on comments or topics raised by the participants during the interviews. This approach provides the interviewer with the freedom to vary the wording and the order of questions, while still ensuring that all topics in the interview guideline are covered [34]. The interviews were conducted by the second author who is experienced in qualitative research and knew the men from the participant observations. The interviews were tape-recorded and lasted between 16 and 56 minutes. Prior to the interviews, the men were informed of the purpose of the study that their statements would be anonymous and that they could choose not to participate in the interviews or drop out without consequences. The interviews were held in locations that ensured privacy. The interviews were fully transcribed, and coded in the software program Atlas.ti.

#### 2.4.4. Analysis

A list of themes was developed inspired by the reviewed literature [34] focusing on the research questions and the theoretical framework. In addition, new topics from the data material arose. A comprehensive list of codes was identified based on the list of themes. The codes allowed us to condense the material and capture overarching themes [33]. Finally, the focus on overarching themes led the attention to themes related to social capital and the themes of well-being. Quotations cited in the paper provide insights of the experiences, motives, and attitudes of the men that are engaged in floorball. The quotations are a representation of the group and have been translated by the interviewer from Danish to English. The anonymity of the interviewees was ensured using pseudonyms, and any identifying information was removed.

## 3. Results

### 3.1. Statistical analyses

#### 3.1.1. Group differences at baseline

The Mann-Whitney test revealed no significant differences between groups for age or the subscales in SF-12 and HADS before the intervention. The results are presented in Table 1.

**Table 1.** Baseline differences.

	*Floorball*	*Petanque*	*p-value*	*Effect size*
Age	69.00 (3.24)	71.07 (3.99)	0.171	−0.23
SF-12 physical health composite score	53.12 (3.63)	54.05 (2.82)	0.435	−0.13
SF-12 mental health composite score	56.18 (5.84)	56.48 (3.63)	0.819	−0.04
HADS anxiety	1.29 (0.34)	1.28 (0.30)	0.891	−0.02
HADS depression	1.23 (0.33)	1.32 (0.31)	0.237	−0.20

Note: Values are reported as means (SD). Effects size is reported as *r*-value.

#### 3.1.2. Intervention effects

The linear regression revealed that there were significant differences between the floorball and the petanque group in development of the SF-12 mental health composite score, as well as the HADS subscales anxiety and depression, from before to after the intervention. It seems that the floorball group developed better in these three subscales (Table 2).

**Table 2.** Linear regression results – group effects on development in health outcomes.

	*Descriptives*				*Regression results*			
*Outcome*	*Time*	*Floorball*	*Petanque*		*F (df1, df2)*	*R^2^*	*B (SE)*	*p-value*
Physical health	Pre	53,12 (3.63)	54.05 (2.82)		0.84 (2,34)	0.05	1.01 (1.52)	0.513
	Post	52.09 (4.06)	51.33 (5.24)					
Mental health	Pre	56.18 (5.84)	56.48 (3.63)		10.61 (2,34)	0.38	3.14 (1.24)	0.041
	Post	57.59 (3.85)	54.59 (5.11)					
Anxiety	Pre	1.29 (0.34)	1.28 (0.30)		11.76 (2,34)	0.41	0.21 (0.11)	0.045
	Post	1.20 (0.24)	1.40 (0.44)					
Depression	Pre	1.23 (0.33)	1.32 (0.31)		14.59 (2,34)	0.46	0.15 (0.08)	0.048
	Post	1.14 (0.16)	1.34 (0.41)					

Note: Descriptives are reported as means (SD). All B-values have been corrected to positives for ease of understanding. They all indicate that the floorball groups developed better quality of life and psychological health

### 3.2. Interview Findings

#### 3.2.1. Experiences with floorball—common background, inclusion and enjoyment

##### (1) Common background

When the men were asked about their experiences with the floorball training, they compared their experience with previous attempts to engage in a physical active lifestyle. As a part of the men's sports biographies many of the men have previously had negative experiences with training in a fitness centre or in individual physical activity. In a Bourdieusian sense, an individual's experiences with sport can be characterised as habitus. Bourdieu describes habitus as a set of”…dispositions, reflexes and forms of behaviour people acquire through acting in society. It reflects the different positions people have in society” [35]. When expressing their view on and experiences of physical activity, many of the interviewees revealed that they felt odd or excluded from the activities in fitness centres that attract a younger audience than themselves, or in senior programmes that to a stronger degree seem to appeal to women. One of the interviewees described his experiences as follows:

“At some point, you want to do something with a little more ‘power’, something that is a bit more exciting, a bit more masculine, I must say (...). I was tired of that old woman gymnastics” (Henry, 68).

In contrast to these earlier experiences of exclusion, the interviewees mentioned several things that made them feel included in the team and attracted to floorball. One element was the fact that the group only consisted of men age 65+, an aspect that the interviewees emphasized as positive. One interviewee expressed his experience of cohesion by comparing it with relationships that were established in their youth: “It's a bit like being army buddies, but just in a much, much later age” (Hans, 71). The men found it very beneficial to train in a group with participants of the same gender and age. The importance of being “at the same stage in life” was highlighted by several interviewees. One phrased it like this:

“I felt that it was like meeting some old school friends (...), it was very encouraging really that we almost had some common background, although we have lived many different lives, then we have after all lived in the same time period (...). There was something that seemed very alluring from the start. I hadn't thought of that as a possibility before” (Anders, 72).

The fact that the men shared a common background was perceived as more important than the differences in their subjective habitus.

##### (2) The inclusive and competitive game of floorball

The interviewees also emphasized the importance of floorball being an activity that none of the men had prior knowledge of, meaning that the men hadn't negatively experienced a significant difference in the level, competences or technical skills:

“We start from scratch all together (...). There is none of us who are world champions in this game, because there is no one who has played much before. Whereas, if you come in a football club, then there is always somebody who is better than others. And I think it has in any case been the contributory factor to why it has been so easy” (Steen, 69).

The challenge of creating an inclusive and enjoyable environment when skills and competences differ, was—as the quote above shows—perceived as a barrier that have kept some of the men from starting or resuming previous sport and exercise programmes. The fact that none of the men were skilled in floorball was an inclusive element that positively influenced the interviewees' perception of floorball.

Several men have expressed that it has been motivating to experience a development in skills, both personally and as a team. It is particularly the competitive element, inherent in the nature of team games, which has been mentioned as a motivating aspect of floorball. This is highlighted as a difference between floorball and individual physical activities:

“You show up to win. You can't change that. No one wants to walk around and think it is fun to lose on purpose. It bothers you. They will win. Every time. And it gives a little extra. ‘Okay, now that damned guy has scored twice, he shall not be allowed any more.’ So you keep an extra eye out for him, and you start to push a little. And one cannot do that in the gym, I can't go and push the guy next to me off his bicycle” (Tommy, 69).

It seems that the competitive element inherent in team games is a motivational factor, not commonly found in the physical activity opportunities available to older men.

##### (3) Enjoyment

The interviewees of this study consider floorball as a form of play that is fun and generates a sense of well-being:

“I must admit, I am incredibly surprised. I had thought that I would be glad to have moved my body, but that it would be so good mentally, I did not expect that in any way (...). I had pulled myself together to go to the gym, I know I would. That's not it. But it hadn't been fun. I do not believe that (...). You'd just go [and exercise], so you can drink a little bit of red wine and eat some good food (…). Whereas here [floorball], it's been a joy. It's almost like if you broke your leg, you would come here anyway and just stand and shout ‘Come on!’” (Benny, 67).

It seems that the men found floorball to be enjoyable and exciting, in contrast to earlier physical activities, that were seen as boring:

“You can't go to gymnastics, it gives me nothing, even though I am an old gymnast. You can't find a place where there is gymnastics that is suitable for old age” (Jakob, 71).

They describe physical activity as exhausting or boring work, which some of the men mention as a barrier to their engagement in physical activity. Contrary to these experiences, the interviewees describe floorball as fun and pleasurable, and something that they were looking forward to with enthusiasm, excitement and joy.

#### 3.2.2. Positive consequences of floorball—improved well-being and social capital

##### (1) Improved well-being

The majority of the interviewees named the aim of improving fitness as their main motive to begin floorball training. The physiological effect of the training was tested during a health check conducted prior to and after the 12-week intervention. The fact that the men could “follow” their own physical development seemed to have been the initial gain of attending floorball training.

Although the interviewees did not know of their personal results from the health check at the time where the interviews were conducted, they expressed that they looked forward to hearing about their performance. Aside from the outcome of the health check, the interviewees talked about the physical and mental improvements they had experienced in their everyday lives as a result of the training. It was especially an increased sense of well-being and quality of life as well as a feeling of improved physical health that were expressed as the main result of the intervention:

“I haven't really had that experience since I was young, that you've used your body. You feel fantastic afterwards (...). I had not thought that it would give that kind of energy. So in that way, I will certainly continue, one way or another, even if I have to arrange it myself” (Benny, 67).

The fact that the men experienced fewer physical and mental limitations was stated by the interviewees as a factor that their significant others also have noted and appreciate: “My wife says it too, she thinks she can feel that I am in a little bit better mood” (Tommy, 69) or as Benny describes his son to have noted: “I have become much more... – it sounds intense, but my youngest son, he says ‘it is almost like you are a socially minded human after all’” (Benny, 67).

##### (2) Social capital

As previously underlined, the men experienced the atmosphere on the team as unique because they were of the same gender and age. This created a kind of cohesion between the players and the social capital was created as an implication of that:

“It's actually fun, for like a start, you could say that we were obviously very heterogeneous in the way of [working in] different industries, different lives and in general. But anyway, then there is, all of the sudden, these things that make you have a lot of common denominators” (Benny, 67).

According to the statements in the interviews, the men experienced a high degree of solidarity and group cohesion, in particular through the fun and joyful experiences on the pitch: “We have great fun together. You could not know that in advance. I think I'm, in general, a little bit suspicious of the social, so it's such a positive surprise that it has been so lovely” (Anders, 72).

The social relations seem to not only develop on the pitch during the interaction in the floorball training, but the social relationships have eventually grown to exist off the training pitch as well:

“I think as we get further in the process, we get more and more socially involved and we are also beginning to talk about what one actually did [workwise] (...). The unity that actually has happened between us. That we have now, after these 12 weeks, begun to talk about many other things to each other than when we started up, where we were more cautious (...). It seems to me, that it [the floorball] has given us something good. You could say it's a better unity that has come out of it” (Roland, 69).

It appears that the men established new social relations, and hence has increased their social capital, during the floorball intervention. The social environment established beyond the training contributes to the team spirit on the team, and it seems that the group of floorball players, because of their “membership” on the team and development of social networks, share a set of values, norms and certain practices in the training setting. Many of the interviewees appreciated that some of the most engaged team members took initiative for a Christmas dinner, for example, which added to the feeling of “being part of something” and belonging to the group.

Consequently, the floorball training was an important source of social capital for the men. It was by many of the interviewees highlighted as an anchor contributing to a meaningful everyday life and several of them compared the team with the community of colleagues before their retirement:

“The difference from being retired and being at work is that you are not part of a community anymore. I could feel that going to the training had a positive effect similar to going to work because then there were three days a week when I returned to the same group” (Tage, 76).

This “anchor” seemed to exist because of the combination of the enjoyable exercise and the social networks that had become crucial to the players' continued participation throughout the 12 weeks.

When the interviewees were asked if these networks had grown into personal relations with meeting outside of the weekly training sessions, the majority of the interviewees expressed that the friendships were restricted to the training sessions:

“They are surprisingly sweet and lovely guys all together. For the time being, there is no one I would like to meet with privately, but I am looking forward to being with them every time. They are damn nice and positive, and we are in the same boat with respect to age and weight class many of us, so I think that's fine” (Henry, 68).

Taken together, it seems that being part of the floorball group created social capital despite differences in habitus, but through the similarities in gender and age, as well as through the enjoyment of and interaction during the floorball activities. It also seems that this social capital is closely tied to the floorball context.

## 4. Discussion

In the present study, the two purposes were to (1) investigate the differences between floorball and petanque in effect on health status and psychological health in untrained men aged 65 or older; and (2) investigate possible reasons for the differences. Overall, we found that (1) floorball, compared to petanque, produced desirable effects for health status and psychological health; and (2) the homogeneous group of men above age 65 created social relationships on and off the floorball pitch, which was expressed as a key factor for the men's positive experiences in the 12-week floorball intervention.

In the SF-12 mental health composite score, the floorball group improved to a larger extent than the petanque group. It seems that the combination of social interaction and physical activity is more beneficial to mental health than social interaction only. This is not surprising, as research suggest that physical activity is beneficial to mental health [16],[17],[36]. However, since some studies suggest that physical activities with different levels of interaction provide similar positive effects on mental health, it is possible that the physical activity yields the primary effect on mental health. This oculd also be the case with the HADS subscales measuring anxiety and depression—that the physical activity is the primary cause of the change in psychological health.

However, it is also possible that the nature of the game floorball yields higher quantity or quality of social interaction between the men, and this is what contributed to the better development in psychological health. Nielsen et al. [21] found in their study that the interactions in football contributed to the experience of higher quality of life, and the results of this study can be explained in a similar way.

Turning to the interview findings, it seems that it was important for the men that they had common background and shared the fact that they are retired. Scholars have argued that it is especially the transition from working life to retirement that is vital in terms of promoting a physical active lifestyle [37],[38]. The interviewees voiced that the importance of “being at the same stage in life”, more specifically, sharing characteristics such as age, gender and (more or less) an equal knowledge and competences within floorball, have made the men to describe the floorball environment as warm and inclusive. This finding is in line with Krustrup et al. [39], who found that exercise training that is conducted in homogeneous groups in relation to gender, age, level of experience and interest in the activity has the potential to form networks and social capital. This may be even more vital to men, since they are less physical active and socially engaged than their female counterparts [25].

Some interviewees expressed that the floorball training was an “anchor” that fill in a gap they have experienced as retiree, as the training create a daily routine similar to work-life and a social community comparable with having colleagues. In line with this, Barnett, Guell, and Ogilvie [37] describe how a sporting leisure environment can compensate for “retirement-related losses” and a way for older adults to develop new routines.

From a Bourdieusian perspective, interacting with one another in itself may not purely be positive. It is the fact that one experiences a feeling of group membership [23] and is met with recognition, respect and communality [23],[40], that social networks are dependent on, and that may develop into increased social capital. Similar findings were found amongst this group of men, who expressed that they felt respected and recognized as a “member” of the group, belonging to the team despite level of technical skills in the floorball activity.

In addition, the interactive and inclusive nature of floorball contributed to the development of social capital, a result similar to the findings of Nielsen et al. [21], who investigated older men's experiences with football. According to the statements of the interviewees in the Nielsen et al. study, the inherent element of interaction on the pitch, and the physical and competitive nature of team sport have slowly generated into social relationships off the pitch. Crossley characterises networks developed through physical activity as: “friendship spill over beyond the gym” [31]. Like Crossley's finding, the interviewees in this study also described the physical activity to be the main driver for the social networks that have been established. It has earlier been acknowledged that supportive social relationships built as a result of an experience of sport engagement is a contributor to adherence to a physical activity [41]. A high level of social capital seems to be associated with a higher level of physical activity, due to the increased social connectedness and lower levels of social isolation [42].

On another note, it is crucial to remember that a “dark side” to social capital and social relations may also exist, where individuals who are not part of a group feel excluded from certain norms and values that are practiced in a particular setting [35]. In relation to sport and exercise in old age, this exclusion may be expressed as negative experiences such as frustrations [43], fear [44] and exclusion [21]. Amongst some of the men, it was acknowledged that commercial fitness centres or programs for seniors with a feminine “touch” had given the interviewees negative experiences of physical activity, where they felt excluded or odd by not sharing similar interest or norms.

Lastly, another cause of adherence is enjoyment. According to Nielsen et al. [21], sport and exercise activities that are fun and pleasurable have potential for a continued partaking in exercise. These findings concur with Kirby and Kluge's [41] results, who found engagement in sport during older adulthood to aroused feelings of excitement. Whilst the interviewees of this study recognize the physiological benefits of participating in sport and exercise in old age as a way of coping with aging, they express the enjoyment, fun and pleasure to play a crucial role in their participation and motivation in the intervention.

All in all, it seems that floorball is an activity well suited to older men for a number of reasons: (1) it increases physiological health and functional capacity [14]; (2) it improves health status and psychological health; (3) it establishes networks on and off the pitch that leads to increase social capital; and (4) it seems to be a motivating activity for older men due to the fact that they are physically active in homogeneous groups with like-minded men both in terms of level of knowledge and competences in the floorball activity as well as where they find themselves in life.

### 4.1. Limitations

This study has a few limitations that should be mentioned. First, the two participant groups were quite small, making our results prone to random error, and to type II errors. However, since our quantitative results and qualitative findings are in congruence, we consider our conclusions to be valid. Second, although floorball and petanque are easy to compare in terms of intensity level, since petanque is an activity with a negligible level of physical activity, the difference is not as clear in terms of social interaction. Petanque also includes social interaction, making it harder to attribute the better development in the floorball group to the amount of social interaction. Furthermore, we argue that there are more social interactions during floorball, but do not substantiate this claim. Again, leaning on earlier research, it seems fair to speculate that the amount of social interactions is higher in floorball because of the amount of physical “interaction” that exists on the pitch as an inherent aspect of team games. Third, we chose to only perform interviews with the floorball players because the team dynamic and group cohesion of floorball were an interest of ours, and hence, we have no grounds for comparing experiences of the two groups. However, we believe that the interview findings can shed some light on why the floorball group developed better than the petanque group in the questionnaire variables. Fourth, no follow-up data collection has been conducted, giving us no possibility to investigate if our findings change over time, or if there is a group difference in adherence over time.

### 4.2. Perspectives

The findings in this study have several implications for future physical activity interventions whose aim is long-term commitment to physical activity for older men. Floorball appears to be a suitable activity for older men, as it seems beneficial for both physical and psychological health, and seems motivating for older men, a target group that in Denmark has trouble with participation in physical activity. First it is important to remember that older men prefer a homogenous group in terms of gender, age, and point in life to ensure a fertile ground for development of social capital. Second, it is advisable to introduce activities that are based on playful team activities such as ball games [45] in municipalities' health centres and other venues that specialize in exercise for the older population [25],[46], as these ensure interaction and enjoyment during the activity. These concepts already exist, such as football fitness, football training that is designed for health and fitness (instead of competition) for new target groups, managed by local football clubs [47]. Third, as many newcomers to physical activity find it difficult to join a physical activity context due to differences in skill, it seems important to ensure an equal challenge for participants, which can be ensured through homogenous skill level. Fourth, as existing physical activity environments can be excluding to newcomers who do not possess the skills to navigate within it, it could be beneficial to help them navigate through this complex system of sports and exercise associations and fitness centres, as well as in the context of the single physical activity group.
